# Radiation Induced Leiomyosarcoma Three Decades After Treatment for Wilms’ Tumor

**DOI:** 10.4021/wjon2010.06.219w

**Published:** 2010-05-19

**Authors:** Muhammad A Khattak, Hilary L Martin, Ganessan Kichenadasse

**Affiliations:** a Oncology Registrar, The Queen Elizabeth Hospital, 28 Woodville Road, Woodville South, Adelaide, South Australia – 5011; b Oncology Registrar, Flinders Medical Centre, Flinders Drive, Bedford Park, Adelaide, South Australia – 5042; c Medical Oncologist, Flinders Medical Centre, Flinders Drive, Bedford Park, Adelaide, South Australia – 5042

**Keywords:** Wilms' Tumor, Radiation induced sarcoma, Surveillance

## Abstract

Wilms’ tumor is one of the most common pediatric malignancies. Survival rates have increased dramatically over the last few decades. This increased survival means that there is an ever growing population of patients susceptible to the late effects of their initial therapy. Survivors of Wilms’ tumor have a substantially higher rate of development of secondary neoplasms compared to general population. We report a case of metastatic radiation induced leiomyosarcoma thirty years after therapy for Wilms’ tumor. This case highlights the need for minimizing the risk of late complications and for close surveillance to enable early detection of these complications.

## Introduction

Wilms’ tumor (WT) is the fourth most common pediatric malignancy [1]. Depending on the stage of disease, chemo-radiotherapy may be used in addition to surgery. Ongoing monitoring for late complications of chemo-radiotherapy is recommended. Survival rates have increased from 20% in the 1960s [2] to 90% with current therapies [3, 4]. There is a 1.6% cumulative risk of second malignant neoplasms in patients treated for WT at 15 years post therapy, with the cumulative risk steadily increasing as time from initial therapy increases [5].

In the WT study, 5,514 patients were followed up. Forty-three patients developed secondary malignancies, with 73% of the solid malignancies occurring in the radiation field [5]. Thirteen out of 43 secondary malignancies found were sarcomas. Of the 13 sarcomas reported, at least 11 would meet the criteria for radiation induced sarcoma (RIS) as proposed by Cahan and modified by Arlen et al [6, 7] of i) previous treatment with radiation therapy at least 3 years prior to the diagnosis of sarcoma; ii) sarcoma arising within the field of previous radiation therapy; and iii) differing histology between the primary tumor requiring radiotherapy and the sarcoma. No patient was reported to have leiomyosarcoma (LMS) as a second malignancy. The prognosis of RIS is poor and worse than the usual prognosis for sarcoma [8]. RIS arise in 0.035-0.2 % of all irradiated patients [9].

We report a case of metastatic post radiation LMS presenting almost three decades after the initial treatment of childhood WT. On review of the English literature there are only two reports of leiomyosarcomas occurring in WT survivors. One presented with rectal leiomyosarcoma, and the second presented with leiomyosarcoma of the descending colon. Neither of the cases had metastatic disease at presentation.

## Case Report

The patient presented to hospital aged 3 with nausea and abdominal pain. Laparotomy showed haemoperitoneum, secondary to haemorrhage from a tumor of the right kidney. Nephrectomy was performed, with spillage of tumor during surgery. No other metastases were present. Histology confirmed Wilms’ tumor. He had no distant metastases. He received a two year course of intravenous vincristine and dactinomycin as well as radiotherapy to the renal bed at a total dose of 27.8 Gy for his stage III Wilms’ tumor. He remained disease-free at his last paediatric oncology follow-up at age 21. He was then referred back to the community to be followed-up by his general practitioner.

In early 2008 at age 35, the patient noticed a right axillary lymph node. He was assessed by a surgeon in April 2008. There was a plan for monitoring this with review again in August 2008 at which time it was still present. A submental node had also developed in the intervening time. He was booked for an excision biopsy but he presented to his local doctor with right upper quadrant discomfort in September 2008. CT scan showed a 39 x 71 mm retroperitoneal mass in the right para-aortic region in the field of his previous radiotherapy. It also showed multiple liver lesions, pulmonary nodules, and lymphadenopathy. He was then referred to Cancer Clinic for further investigation and management.

**Figure 1:**
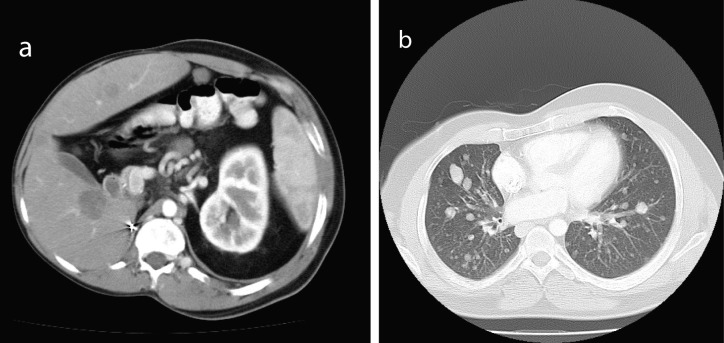
Staging CT showed multiple metastases (a, b).

**Figure 2:**
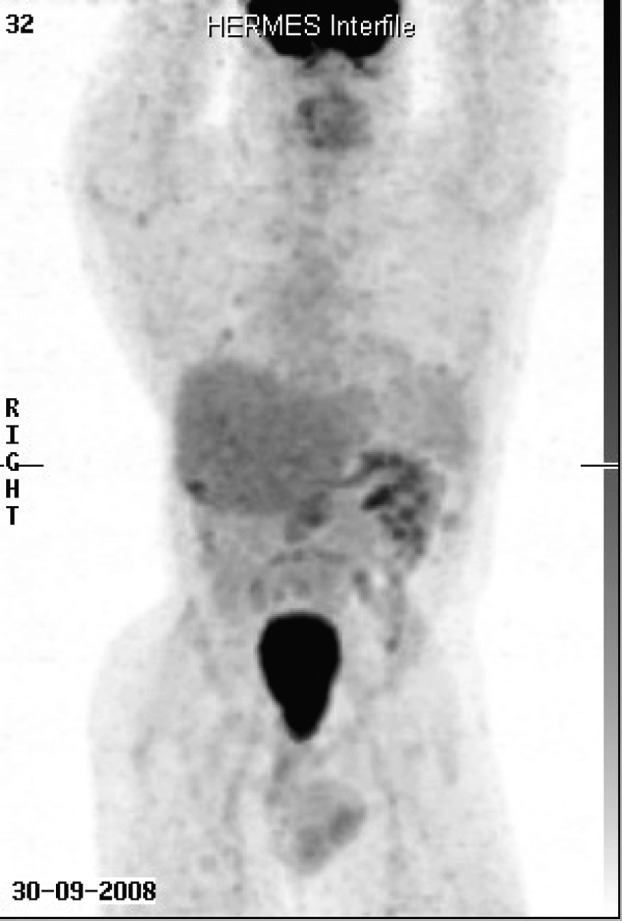
Staging positron emission tomography-CT scan showed uptake in the para-aortic region, the liver, a nodule inferior to the spleen, a nodule anterior to the proximal right latissimus dorsi muscle and variable uptake in the pulmonary nodules.

When first seen at the Cancer clinic, he appeared to be a pleasant, average built man with extensive scoliosis of his spine. He had an enlarged liver and axillary and submental lymphadenopathy. He underwent an excision biopsy of the axillary node. Histopathology was consistent with leiomyosarcoma, with well encapsulated, moderately pleomorphic and mitotically active smooth-muscle tumor. Desmin stain was positive and smooth muscle actin stain showed patchy positivity; AE1/AE3, S100 and C-Kit and EBER were all negative. Staging CT scans were performed which showed multiple metastases (Fig. 1). A staging positron emission tomography-CT scan showed uptake in the para-aortic region, the liver, a nodule inferior to the spleen, a nodule anterior to the proximal right latissimus dorsi muscle and variable uptake in the pulmonary nodules (Fig 2). The investigations confirmed a diagnosis of RIS of LMS subtype, with dominant mass in the previous radiotherapy site and extensive metastases almost three decades after his initial treatment. He was offered palliative chemotherapy, but he declined and went onto having various alternative therapies and finally succumbed to his cancer nearly 12 months after the diagnosis.

## Discussion

There has been an increase in the number of patients with RIS over the last few decades, perhaps due to the increased use of aggressive chemoradiotherapy with radiosensitizers such as doxorubicin proposed to play a role in this [10]. Increased survival rates from the primary malignancy may also be a contributing factor. None of the patients in the WT Study developed LMS, and only two other cases of secondary LMS in patients with WT are reported in the literature [11, 12]. Both these cases, as well as most of the cases of radiation induced leiomyosarcoma reported, presented with localized disease.

Patients undergoing treatment for WT under the current protocols are receiving less radiotherapy than earlier protocols. Patients with stage III WT now receive a total of 10.8 Gy under NWTS-5 or a total of 15 Gy if treated via SIOP93-01 protocol, both significantly less than the 27.8 Gy that our patient received. However, under current treatment protocols, such patients receive doxorubicin in addition to vincristine and dactinomycin. Concerns have been raised in the literature that doxorubicin acts as a radiation sensitizer and may increase the risk of radiation induced sarcomas [5]. The National WT Study found that doxorubicin use in addition to radiotherapy leads to significantly more second malignant neoplasms (SMN) than expected. Each additional 10 Gy of radiation increased the risk of SMN by 43% while each additional 10 Gy of radiation in the presence of doxorubicin increased the risk by 78%. Thus the risk of RIS still remains relevant to the WT patients undergoing treatment as per current guidelines of lower radiation dose.

One of the major issues raised by our case is that of the long term follow-up. With WT survival rates now greater than 90%, there is an ever increasing ageing population of patients susceptible to the long-term effects of the initial therapy for WT. The longer the post therapy survival, the more likely the development of secondary neoplasms, but also the more difficult it is to maintain follow-up. The Children’s Oncology Group Long-Term Follow-Up Guidelines for Survivors of Childhood, Adolescent and Young Adult Cancers [13] outlines follow-up required for patients based on treatment received, which includes yearly examination of skin and lymph nodes associated with irradiated area for secondary malignancy.

The reported patient was not followed-up annually after discharge to his community practitioner. However, even had he undergone regular follow-up as per Children’s Oncology Group guidelines, his first clinically evident sign may have still been his palpable axillary lymph node, with metastatic LMS. Given that 73% of the secondary solid malignancies reported in the WT group study developed in the radiation bed as in this reported case [5], an area that is not necessarily easy to detect a mass at an early stage, the question of routine surveillance imaging in long term survivors is raised.

In summary, this case raises the important issues of survivorship and risk of late complications in patients treated for WT and follow-up. Further cohort studies are required to address questions of whether surveillance imaging should be performed in long term survivors, particularly to the radiation bed. Further studies are also required to address the observational data from the WT study regarding the use of doxorubicin and increased risk of second malignancies when used in combination with radiotherapy. Patients and their families need to be educated regarding long term effects of their chemoradiotherapy and the importance of lifelong annual follow-up.
